# Inkjet-Printed 3D Sensor Arrays with FIB-Induced Electrode Refinement for Low-Noise Amperometric Recordings in hiPSC-Derived Brain Organoids

**DOI:** 10.1021/acssensors.4c03740

**Published:** 2025-08-21

**Authors:** Inola Kopic, Hu Peng, Sebastian Schmidt, Oleksandr Berezin, Senyao Wang, Gil G. Westmeyer, Bernhard Wolfrum

**Affiliations:** † Neuroelectronics, Munich Institute of Biomedical Engineering, School of Computation, Information and Technology, 9184 Technical University of Munich, Hans-Piloty-Str. 1, 85748 Garching, Germany; ‡ Neurobiological Engineering, Munich Institute of Biomedical Engineering, 686517 TUM School of Natural Sciences & TUM School of Medicine and Health, Boltzmannstraße 11, Garching 85748, Germany; § Institute for Synthetic Biomedicine, Helmholtz Munich, Ingolstädter Landstraße 1, 85764 Neuherberg, Germany

**Keywords:** microfabrication, microelectrode arrays, neuroelectronics, extracellular recording, organoids

## Abstract

Understanding the functional connectivity and behavior of 3D cell cultures and organoids requires monitoring electrical activity across multiple planes. However, traditional planar microelectrode arrays (MEAs) are limited to surface recordings and struggle to capture signals from deeper layers. Additionally, current fabrication methods face challenges such as prolonged production times and limited design flexibility, which hinder the development of high-precision 3D electrode arrays and affect the quality of cell-electrode coupling. To overcome these obstacles, we introduce a new approach that integrates inkjet printing with focused ion beam (FIB) milling and electrodeposition, resulting in highly customizable 3D MEAs. The FIB milling enables the creation of precise electrode openings at predetermined locations, which is essential for selective recordings within the tissue. The MEAs, fabricated on glass substrates, incorporate high-aspect-ratio (up to 44:1) electrode structures with heights up to 1 mm, a pitch of 500 μm, and electrode openings of 3 and 6 μm, providing the necessary resolution for targeted measurements. Impedance and noise characteristics (down to a root-mean-square of (RMS) noise of 0.2 pA) for amperometric measurements were assessed in dependence on the electrode size. We demonstrate the effectiveness of these 3D MEAs by recording electrophysiological activity from hiPSC-derived cortical organoids (age: 24 month) both in situ and after 10 days of cultivation of the organoid directly on the MEA. This approach facilitates in vitro studies of neural activity in organoids and holds promise for high-throughput, selective amperometric analyses in normal and pathologically altered conditions.

## Graphical abstract

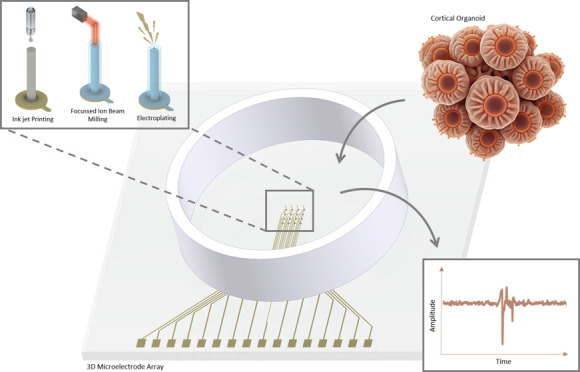

To advance the study of neural networks without reliance on animal models, a range of in vitro models has emerged, progressing from 2D cultures to intricate 3D multicellular structures. A major advancement has been the development of organoids, which self-organize and contain diverse cell types that simulate the organization and communication patterns seen in brain tissue.^1–3^ Such structures allow the study of neural connectivity, which can be examined by recording electrical signals using MEAs. In the past, MEAs have been widely used in vitro with various cell models, traditionally incorporating planar 2D electrodes or, more recently, high-density MEAs.^4–8^ However, with 3D spheroid and organoid cultures emerging, there has been an increased need for MEAs that function effectively within 3D environments, leading to the creation of stretchable and flexible MEAs suited for these applications.^9–11^

Innovative MEA designs now include structures that wrap around, contain, or serve as a supportive structure for 3D cellular clusters, enabling external signal recording.^12–17^ Alternatively, some approaches have incorporated mesh-like electrodes embedded into organoids to facilitate inner-structure signal monitoring, though these setups do not fully address multiplanar signal acquisition or allow easy removal postmeasurement.^18–20^ To address these limitations, pillar-based MEAs have emerged as a minimally invasive option that can achieve targeted, in-depth recordings.^21^

Advanced methods for 3D MEA fabrication include cleanroom processes integrated with electroplating, two-photon lithography, and modified bonding techniques.^22–24^ Yet, producing high-aspect-ratio pillars with micron-scale electrode openings remains challenging. Additive manufacturing methods, such as aerosol and inkjet printing, present promising alternatives to the more elaborate fabrication approaches.^25–29^ In particular, inkjet printing offers advantages in terms of customization and scalability for high-throughput applications, making it a feasible choice for fabricating 3D MEAs. In previous studies, inkjet printing was used to produce silver-based pillar MEAs, which were subsequently coated with platinum and gold (Au).^30^ To address challenges with the insulation of the 3D structures and silver ion leakage, a parylene passivation layer was introduced that was subsequently removed at the tip using a laser-based ablation process.^31^ While applicable for in situ cortical organoid recordings, laser-based ablation processes typically lack precise control of the electrode size and position on 3D electrode structures.

To tackle these challenges, we established a streamlined process that integrates maskless lithography, inkjet printing, FIB milling, and electroplating. Our approach enables the production of high-aspect-ratio (44:1) 3D microstructures on rigid glass substrates with precise control of the electrode size, providing a user-friendly and stable platform for in vitro applications. FIB milling is widely utilized in materials science for structural and compositional analysis and in the fabrication of nanoscale features and devices due to its accuracy, controlled material removal, and compatibility with a wide range of materials.^32–35^ Using this method, we fabricated electrode openings in the micron range (3 and 6 μm in diameter) with high control over size and placement. We analyzed the impedance and noise during amperometric recordings in dependence on the electrode size. The effectiveness of our fabrication method was validated by directly measuring electrophysiological signals from cortical organoids derived from human induced pluripotent stem cells (hiPSCs) on-chip in a rapid in situ manner, preventing damage to the organoid.

## Results and Discussion

### Fabrication of Pillar Microelectrode Arrays

The proposed fabrication process is schematically illustrated in Figure 1A. Generally, the 3D MEAs consist of several components (Figure 1B) and are fabricated in sequential steps as follows. The first step in creating a complete MEA involves spin-coating a thin layer of photoresist onto the glass substrate (Figure 1A­(i)). Using glass as a rigid substrate offers several advantages: it facilitates easier handling (e.g., with tweezers, as seen in Figure 2B) by minimizing the risk of bending or damaging the device, and its transparency enables direct visualization of the organoid or tissue when placed on the chip under an inverted microscope.

**Figure 1:**
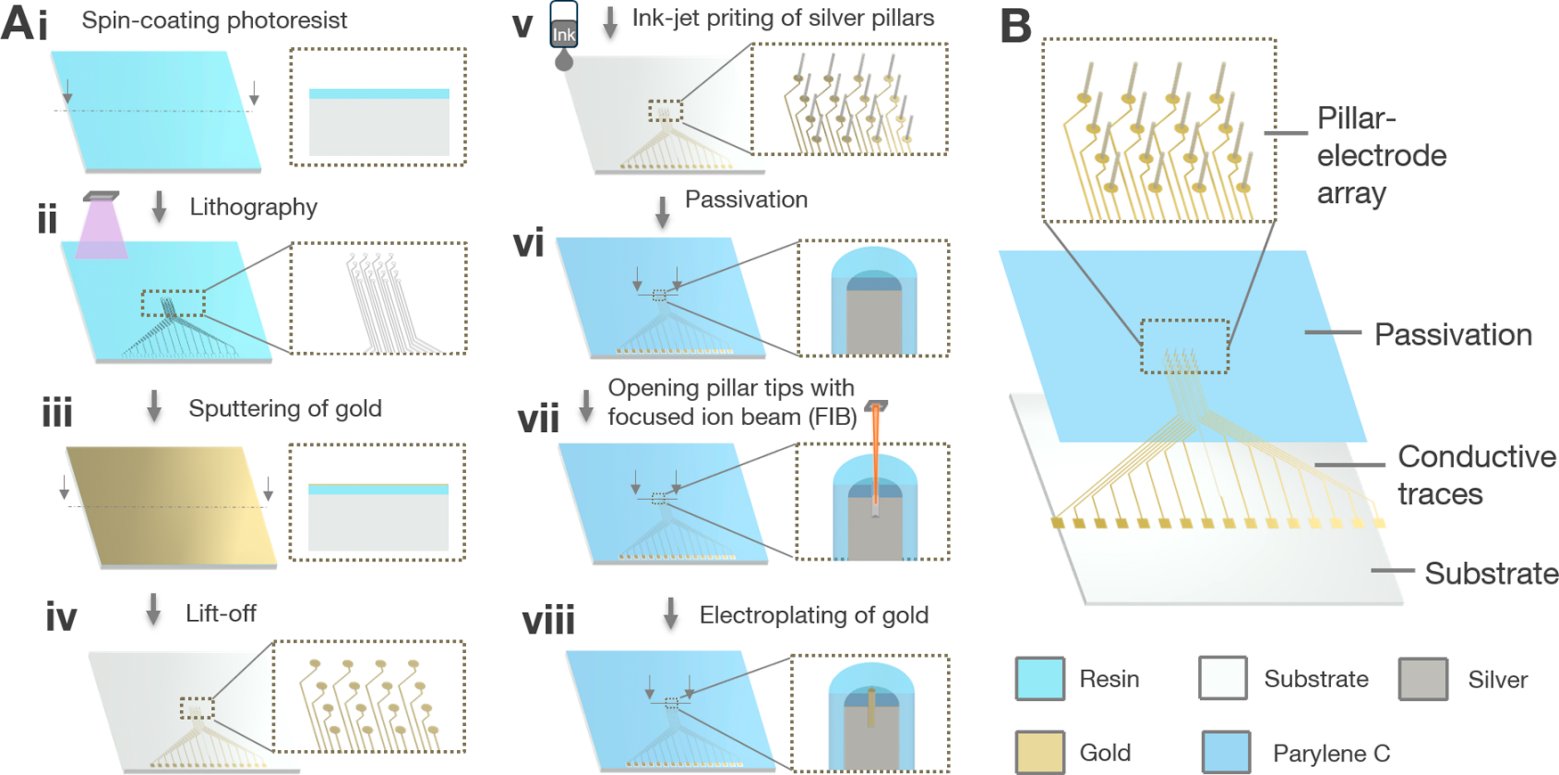
Fabrication process of 3D MEAs. (A) Schematic fabrication approach for creating high-aspect ratio pillar arrays combining maskless lithography, ink-printing, FIB, and electrodeposition of gold with different tip diameters. (B) Illustration of the various layers of a 3D MEA.

**Figure 2:**
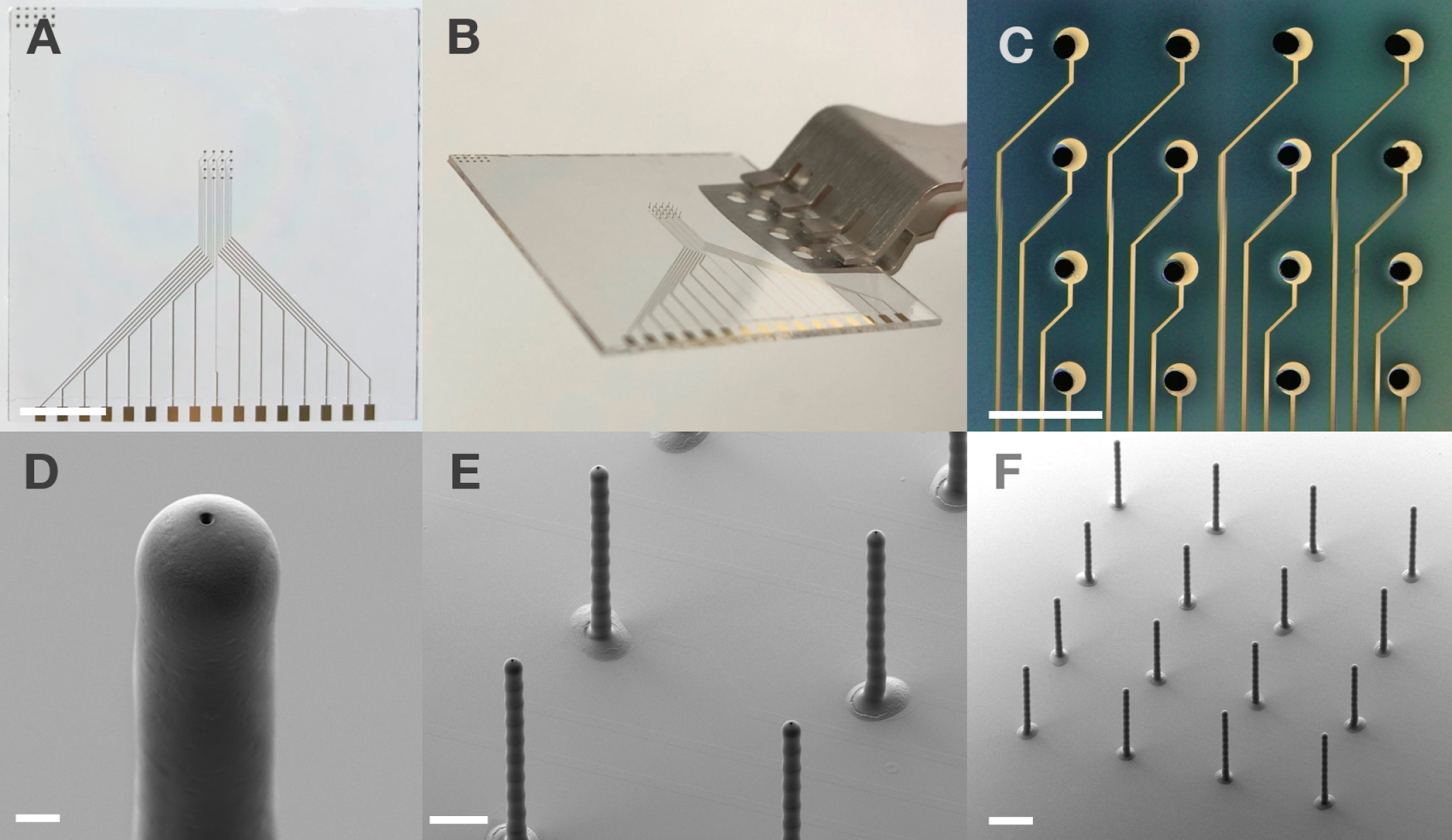
Images of 3D MEAs and individual pillars. (A) Top view picture of a 3D MEA with gold feedlines and electrode pads after passivation with pillars reaching up to 500 μm in height on glass after competition of the fabrication process. Scale bar representing 5 mm. (B) Side-view picture of a MEA with 3D structures of 350 to 500 μm height on glass after gold electroplating held with a tweezer. (C) Top view microscopic image of a 16-electrode array with 3D electrodes, 480 μm in height, on the round golden electrode pads of a MEA with a pitch of 500 μm. Scale bar indicating 500 μm. Scanning electron microscopy image (SEM) of (D) an individual pillar with an opening diameter of 3 μm with a pillar height of 470 μm, (E) 4 neighboring pillars with opening diameters of 6 μm and pillar heights up to 500 μm, (F) a 3D MEA before FIB milling with 16 electrodes of up to 500 μm pillar height. All SEM images were acquired using an electron beam acceleration voltage of 3 kV. The scale bars shown have a length of 10 μm, 100 μm, and 200 μm (D–F), respectively.

A maskless aligner was employed to pattern the layout into the photoresist. The UV LED source selectively exposes only targeted areas of the film, eliminating the need for a photomask. (Figure 1A­(ii)). Ensuring good adhesion between the thin metal layer and glass is crucial for subsequent process steps; hence, a thin layer of titanium (Ti) was presputtered on the glass to create a strong bond between the gold layer and the glass substrate (Figure 1A­(iii)). A lift-off technique was used (Figure 1A­(iv)) to remove the excess Ti–Au film, resulting in individually addressable electrodes, as illustrated in Figure 2A. The lithography combined with the lift-off step establishes precise feedlines and electrode pads, which enable individual addressing of the 3D pillars and connection to a readout system. As an alternative, laser patterning could be used to form feedlines and bond pads, as reported in prior work, which can reduce the patterning time for larger samples.^31^ However, in this study, the primary focus was the precise placement of feedlines and the creation of small electrode pads with thin traces. Since laser patterning is constrained by the laser’s spot size (20 μm), maskless lithography was chosen to produce thinner feedlines, ensuring clear visualization of the organoid under an inverted microscope during long-term studies. The thin feedlines and round electrode pads are shown in Figure 2C.

Following the formation of the 2D MEA layout, silver (Ag) pillars are added to the individual electrode areas through a drop-on-demand (DOD) inkjet printing process (Figure 1A­(v)). In this step, voltage pulses applied to a piezoelectric actuator create pressure pulses that expel droplets of silver nanoparticle ink from the printhead nozzle onto the substrate. Successive droplets are precisely aligned and deposited at specific electrode sites, building up a pillar-like structure, as shown in Figure 2D–F. The diameter and height of these pillar electrodes can be adjusted based on parameters such as the number of droplets, ink properties, and initial droplet–surface interaction. In this study, high-aspect-ratio pillars were produced, with an average diameter of 23 μm and heights ranging from 250 μm to 1 mm. This high aspect ratio of up to 44:1 allows for adaptable recording at different heights in later stages, supporting measurements across a range of biological samples, from small organoids and spheroids to larger tissue sections.

A critical factor when measuring organoids is the ability to address multiple regions accurately from specific locations. This is achieved by insulating the shaft of each pillar and its connecting feedlines while leaving only the pillar tips exposed. To accomplish this, we applied a chemical vapor deposition (CVD) coating of parylene C over the entire MEA, with the contact pads shielded during coating, as shown in Figure 1A­(vi). Parylene C offers mechanical robustness, biocompatibility, and sufficient dielectric properties, making it ideal for biomedical applications. It also provides structural support to the pillars, enhancing stability that is particularly crucial when placing organoids on top, as this prevents the pillars from bending under load. This fabrication approach further prevents issues like underlying air bubbles or contaminants that could compromise MEA performance.^36^

After the entire chip was passivated, the tips of the 3D electrodes were opened using FIB milling (see Figure 1A­(vii)). This process removes the passivation layer from the top of the pillar electrodes with high precision, creating holes at exact, predefined locations, as shown in Figure 2D. The FIB milling technique also allows for controlled removal of the underlying silver layer down to a specified depth. By modulating the milling parameters, the ion dose, and beam current, the size and depth of the openings can be precisely controlled, with diameters and depths ranging from the nanometer scale to several tens of microns. Unlike laser patterning, FIB milling offers substantial advantages in precision and ensures minimal lateral damage. Smaller electrode openings, such as 3 and 6 μm, enable localized recordings of signals from individual cells or smaller tissue clusters in contrast to ∼20 μm openings typically created with a laser-based process.^31^ To evaluate the effect of electrode size on selectivity and signal quality, both 3 and 6 μm diameters were fabricated and tested on the same chip, allowing for a direct comparison of the electrode noise and the acquired signal amplitudes during amperometric recordings. This flexibility in electrode opening size provides the adaptability needed to optimize spatial resolution and signal-to-noise ratio based on the specific requirements of different tissue types.

After milling, the gold layer is removed with a diluted aqua regia solution, which dissolves both the gold and any residual gallium. This ensures the surface regains its nonconductive properties, allowing direct contact between the cells and electrode opening/the parylene C layer, as intended for subsequent analysis.

A key drawback of using silver as the conductive material in MEAs is its cytotoxicity, which can negatively impact cell viability.^37^ To enable long-term measurements, we electroplated gold onto the pillar tips (see Figure 1A­(viii)). This final step aimed to ensure the biocompatibility of the electrodes. The electroplating was carried out using pulsed electrodeposition (PED), which involves alternating deposition cycles for metal buildup and reverse cycles for ion recovery. This technique helps produce a smoother and more homogeneous gold layer by removing surface impurities and hydrogen that could otherwise become trapped.

The deposition was performed at a reduction potential of −1.15 V vs Ag/AgCl, as detailed in the experimental section. The electroplated gold successfully filled the exposed silver pillar tips and formed a domed shape at the top, improving the structural integrity and conductivity of the electrodes. Before and after electroplating, optical and electrochemical characterizations were performed on the 3D MEAs to evaluate the pillar electrode quality and stability.

### Characterization of Pillar Microelectrode Arrays

To assess the quality of the passivation layer, we performed stability testing using chronoamperometry, where a constant voltage is applied to initiate hydrogen reduction on the electrodes, producing bubbles at the openings. This technique allows for the detection of potential pinholes in the feedlines, which would indicate passivation failure. The parylene C coating showed stability, with no pinholes detected along the feedlines, and electrolysis was only observed at the electroplated pillar tip. This confirmed the effective insulation of the passivation layer.

To characterize the diameter of the openings, profilometric microscopy combined with scanning electron microscopy (SEM) was employed. The measured average diameters of the pillar openings for the intended 3 and 6 μm diameter electrodes were found to be 3.1 ± 0.1 μm (*n* = 16) and 6.0 ± 0.1 μm (*n* = 16) (mean ± standard deviation; measured from two individual MEAs), respectively.

To evaluate the electroplating quality and ensure complete coverage of the silver, cyclic voltammetry (CV) was performed in phosphate-buffered saline (PBS). The CV response of both single silver and gold-plated 3D pillars in PBS scanned between −0.4 and 0.8 V at a rate of 50 mV·s^–1^, was compared (Figure 3C). Silver oxidation and reduction peaks typical for silver were observed at 112 ± 2 mV and −117 ± 2 mV vs Ag/AgCl (3 M NaCl). Following electroplating, the gold electrode showed a reduction in the silver oxidation/reduction peaks for both pillar diameters, confirming successful gold deposition.

**Figure 3:**
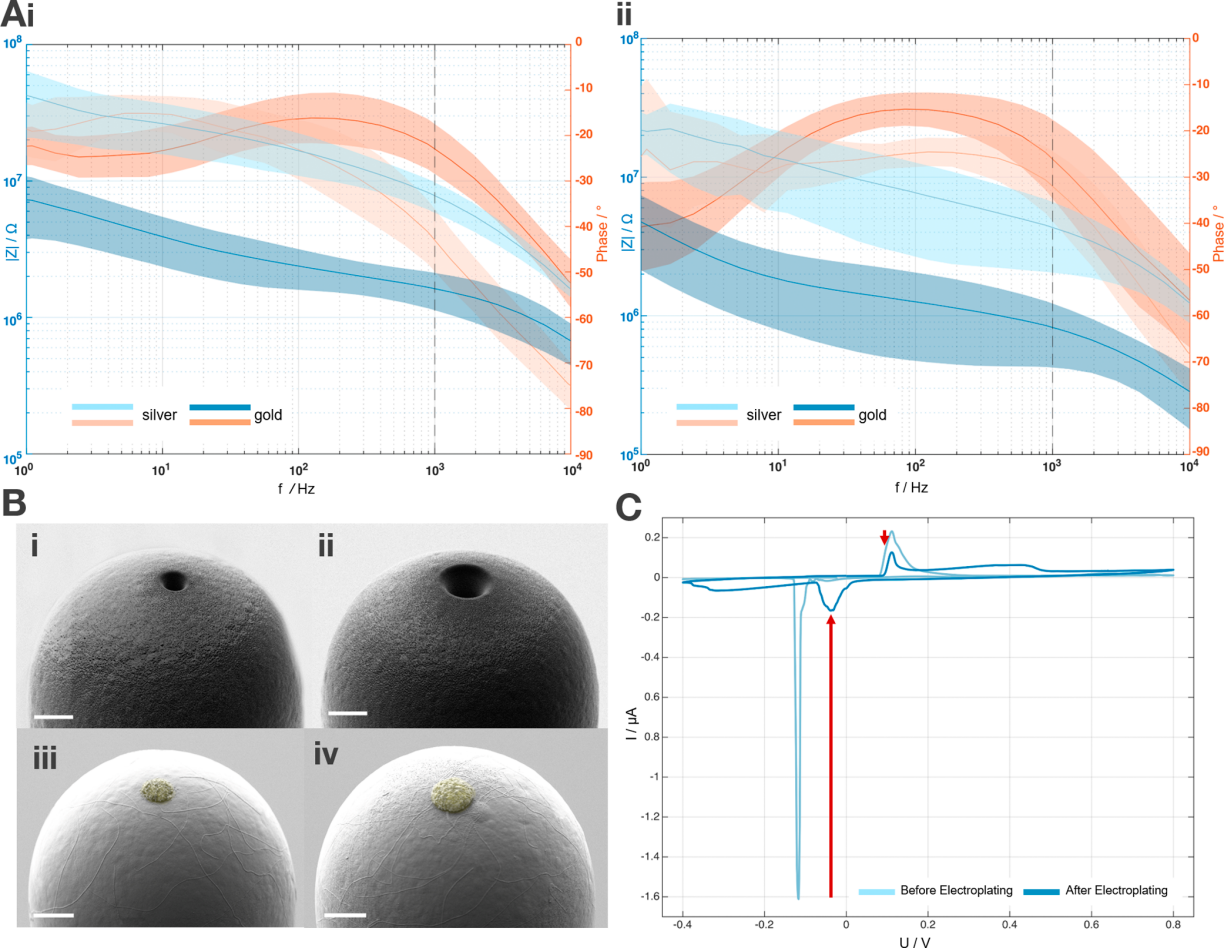
Electrochemical and optical characterization of 3D pillar electrodes after FIB milling and after gold electrodeposition. (A)­(i,ii) The impedance (phase and magnitude) of 3D pillars with 3 μm (i) and 6 μm (ii) openings before and after Au electroplating with −1.15 V vs an Ag/AgCl reference electrode, respectively. The mean and standard deviation (solid line and shaded area, respectively) were calculated across 8 samples. (B)­(i–iv) SEM images of individual 3D pillar tips after FIB milling (i,ii) and after electroplating with −1.15 V vs an Ag/AgCl reference electrode (iii,iv), with openings of 3 μm (i,iii) and 6 μm (ii,iv), respectively. The colored area indicates gold (iii,iv). SEM images (i,ii) and (iii,iv) used an acceleration voltage of 2 kV and 3 kV, respectively. All scale bars shown have a length of 5 μm (i–iv). (C) Cyclic voltammetry plot shows the current as a function of potential for the working electrode (single pillar), both before and after gold deposition. The potential was swept from −0.4 to 0.8 V at a scan rate of 50 mV·s^–1^ against an Ag/AgCl reference electrode. The red arrows highlight the reduction in the silver reduction/oxidation peaks in the samples that were electroplated with gold tips.

For further electrochemical characterization of the individual pillars after FIB milling and gold electroplating, impedance spectroscopy (EIS) was conducted in PBS. A low-amplitude sinusoidal signal of 10 mV amplitude was applied over a frequency range of 1 Hz–10 kHz. The mean impedance of individual pillars with 3 and 6 μm openings before and after gold deposition is shown in Figure 3A­(i) and Figure 3A­(ii), respectively (*n*_3_ = *n*_6_ = 8). At 1 kHz, the impedance for recessed silver-core pillars was 8.3 ± 2.1 MΩ for 3 μm and 4.5 ± 2.4 MΩ for 6 μm openings, whereas the pillars with gold tips showed impedances of 1.7 ± 0.5 MΩ and 852 ± 424 kΩ for the same opening sizes. These results demonstrate that gold electroplating reduces the impedance, likely due to the increased surface area of the gold tips compared to the original silver cores, as shown in Figure 3Bi–iv.

### Pillar MEA Recording of hiPSC-Derived Cortical Organoids

We demonstrated the applicability of 3D MEAs for measuring the activity of hiPSC-derived cortical brain organoids. Cortical organoids that mimic embryonic human cerebral cortex development were generated as described in the experimental section. Most cells in the neural-tube-like structures expressed neuronal precursor (NeuN) and intermediate progenitor (TBR1) markers, developed into layered cortical regions, and differentiated into neurons of both the upper (CUX1) and lower (BCL11B) cortical layers. As differentiation continued, these layers became less distinct, leading to a more uniform distribution of cortical neurons by 24 months (Figure 4A). After this point, no further structural changes were observed, and the neurons matured, forming spontaneous activity patterns within the organoid.

**Figure 4:**
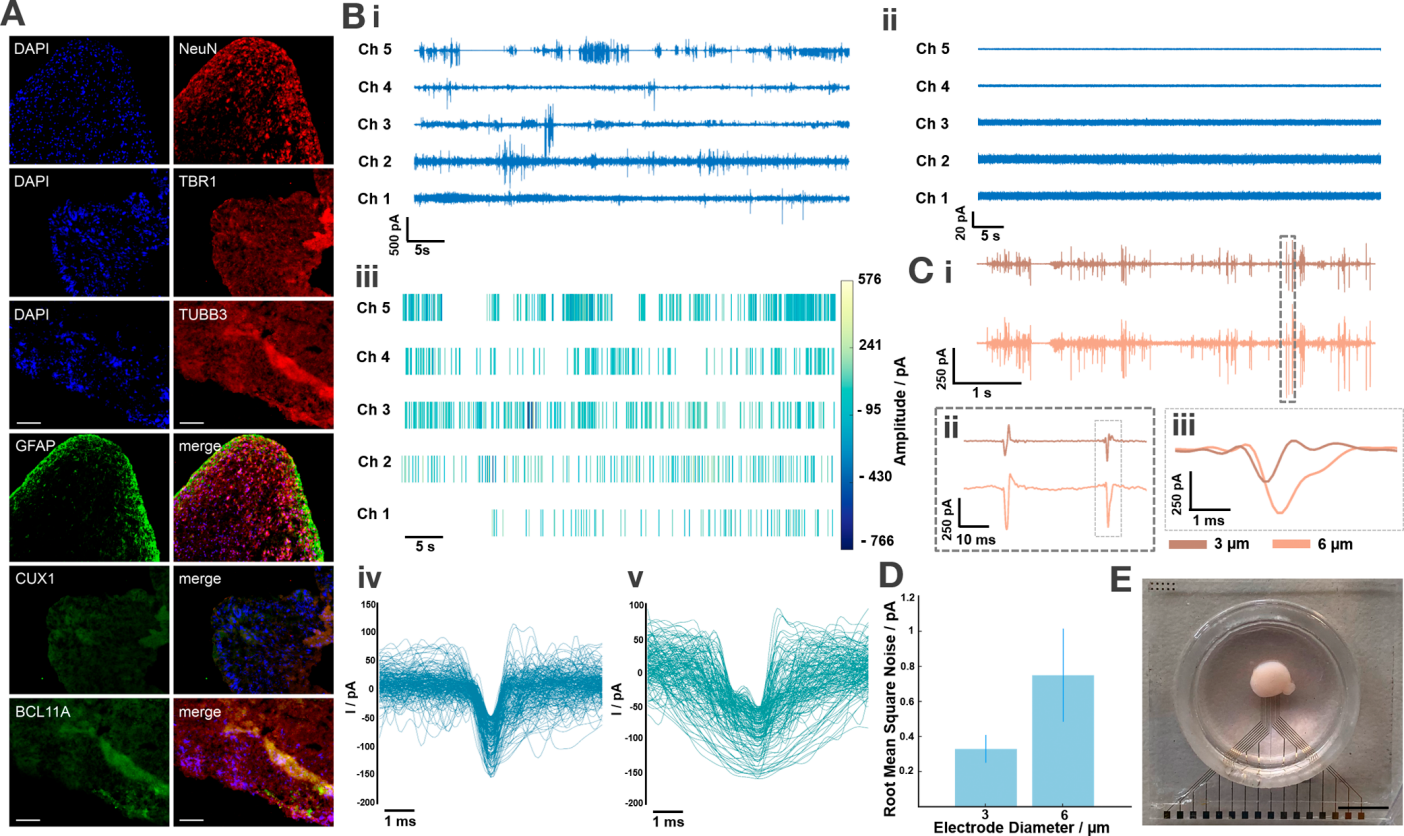
3D MEA recording on hiPSC-derived cortical organoids. (A) Fluorescence microscopy of a 40 μm thick cortical organoid section after recording experiments (DAPI, blue; NeuN, TBR1, TUBB3, red; and GFAP, CUX1, BCL 11A, green). The scale bar in all images has a length of 200 μm. (B) Time traces from in situ recording (i) and postorganoid removal (ii). Channel 1–3 (6 μm) and Channel 4–5 (3 μm) correspond to Au-electroplated pillars with an average height of 471 ± 4 μm (iii). Spike amplitude and temporal occurrence detected from the traces (i) (minimum threshold = 50 pA; *n* = 198 (iv), *n* = 145 (v)) (C)­(i–iii) Time traces of synchronized electrical activity recorded from an organoid (age: 24 months) after 10 days in vitro (i), with a zoomed-in view showing synchronized spikes from two electrodes (3 and 6 μm opening diameters), separated diagonally by 1.4 mm (ii). The overlapping spike profiles indicate temporal stretching and shifting of the signals (iii). (D) Bar plot of the root-mean-square (RMS) noise levels for 3 and 6 μm diameter electrodes, with error bars representing the standard deviation (*n*_3μm_ = *n*_6μm_ = 30). (E) Image of a 24 month-old organoid on a 3D MEA after 10 days in vitro, with 3 and 6 μm tip openings and an average pillar height of 400 ± 3 μm. The scale bar shown has a length of 5 mm.

For this study, two experimental protocols were followed. In both setups, fully matured cortical organoids of various sizes (ranging from 500 μm to 5 mm in diameter) were placed into the well of the MEAs, which featured pillar heights of 250–500 μm, as shown in Figure 4E. Each chip contained eight pillars with 3 μm openings and eight pillars with 6 μm openings, resulting in a total of 16 recording electrodes per MEA. Amperometric recordings were carried out using a custom-built amplifier system. The extracellular signals were filtered with a 60 Hz high-pass and a 1200 Hz low-pass filter to better isolate the spontaneous and synchronized bursts of electrical activity.

For in situ experiments, spontaneous burst activity was recorded from the cortical organoids. Representative traces for 3 μm (Ch1–Ch3) and 6 μm (Ch4, Ch5) openings are shown in Figure 4B­(i) (working electrodes *n*_total_ = 16, signals recorded from 88% of channels during a single recording). To verify that the signals originated from neurons, negative control recordings were performed on the same chip without organoids (Figure 4B­(ii)), revealing no characteristic signals. The root-mean-square (RMS) noise was assessed for electrodes with diameters of 3 and 6 μm, exhibiting values of 0.3 ± 0.1 pA and 0.7 ± 0.3 pA, respectively (*n*_3μm_ = *n*_6μm_ = 30), as shown in Figure 4D. As expected for amperometric recordings, the noise increases with increasing electrode size, in contrast to voltage recordings, where higher electrode impedances generate higher noise levels.

For spike detection, a threshold of 50 pA was applied, and the temporal occurrence and amplitude of the spikes are displayed in Figure 4B­(iii). The average peak-to-peak amplitude for 6 μm electrode channels was 626 ± 62 pA, compared to 350 ± 56 pA for 3 μm electrode channels recorded during the same session. The higher peak-to-peak amplitudes observed for larger electrodes are expected as the signals are capacitively coupled via the electrode–electrolyte interface to the transimpedance amplifiers of the headstage. The organoids exhibited characteristic bursting activity in clusters, which is in line with previous studies indicating that as organoids mature, they tend to show increased bursting behavior.^38^ Our analysis also revealed different spike shapes (Figure 4B­(iv,v)). Specifically, short-duration, high-amplitude spikes were observed, probably originating from single neurons, while slower signals with lower amplitudes reflected compound recordings from multiple neurons.

For the second set of experiments, organoids were cultured on the 3D pillar electrodes of the MEA for 10 days in vitro. On day 10, amperometric recordings of electrical activity were performed, and the signals were analyzed and filtered in the same manner as previously described. Similar to the in situ recordings, the organoids exhibited burst activity. However, the bursts differed from previously spontaneous, random firing to more structured, coordinated bursts, as seen in Figure 4Ci. Synchronized activity was observed across pillars, with 38% of all channels exhibiting such behavior (Figure 4C­(i–iii)). This observed increase in synchronization may be partially attributed to improved cell-to-electrode coupling, which likely developed during the 10 day cultivation period. In Figure 4C­(iii), correlated signals with time delays between individual channels with 3 and 6 μm opening diameters are evident. The signal propagation speed was calculated to be in the range of 1.2 m·s^–1^, determined by the time delay between spikes and the distance between the pillars. Since the exact path of signal propagation is not known, this value should be seen as a lower boundary. The propagation speed is consistent with typical values for electrical signal transmission within neuronal clusters, ranging from 0.5 to 2 m·s^–1^ depending on factors such as the axonal diameter and tissue type.^39^ The spike shapes observed across different pillars in the synchronized channels exhibited a high degree of similarity, with the primary variations being in the amplitude and duration of the signals (compare Figure 4C­(iii)). These findings suggest that the observed activity is likely generated by the same neuronal population or a highly interconnected network. This interpretation is supported by recent studies, which have linked enhanced synchronization with the maturation and development of neural networks within brain organoids.^5,6,40^ As these networks mature, more coherent activity patterns emerge, reflecting the formation of robust synaptic connections and improved intercellular communication within the network, as well as faster signal propagation velocities.

It is well-known that smaller electrodes can selectively capture signals from individual neurons, while larger electrodes tend to recruit signals from a higher number of sources. Furthermore, smaller electrodes provide lower noise levels in amperometric recordings (in contrast to voltage recordings).^41,42^ Here, the difference in noise is evident in measurements obtained without organoids (see Figure 4B­(ii),D) between 3 and 6 μm electrodes. The 6 μm electrodes expose approximately four times the surface area of the 3 μm electrodes, resulting in a lower impedance (see Figure 3A­(i,ii)).

For voltage recordings, a lower impedance results in a lower Johnson–Nyquist noise level

$$S_{v,th}=4\cdot k_{B}\cdot T\cdot Re\left\{Z\left(f\right)\right\}$$

where *S*_V,th_ is the power spectral density of the voltage noise, *k*_B_ is the Boltzmann constant, *T* is the temperature, Re­{*Z*(*f*)} represents the real part of the complex impedance, and *f* is the frequency.

In contrast, for amperometric recordings, the decrease in impedance is reflected in a higher noise level, as expected for a current measurement due to the thermal motion of charges

$$S_{I,th}=4\cdot k_{B}\cdot T\cdot Re\left\{Z{\left(f\right)}^{-1}\right\}$$

where *S*_I,th_ is the power spectral density of the current noise, and Re­{*Z*(*f*)^−1^} represents the real part of the complex admittance (inverse impedance). Yet, for the electrophysiological signals recorded in this study, the advantage of lower noise comes at the cost of lower signal amplitudes, which is caused by the decrease in interfacial capacitance. This might differ for electrochemical measurements, depending on the source of the signal.^43^ For example, the quantal release of redox-active neurotransmitters from individual cells might exhibit similar signal amplitudes, regardless of electrode size, as long as a comparable number of molecules can be oxidized, which would be expected if the release occurs in close proximity to the electrode surface. In the above-mentioned scenario, our 3D microelectrode array could be applied for spatially resolved neurotransmitter recordings within organoids, which should be investigated in future studies.

## Conclusions

We have developed a method for fabricating 3D MEAs, allowing localized recording of organoid activity both in situ and over extended time periods. By integrating maskless lithography, inkjet printing, focused ion beam milling, and electrodeposition, we produced high-aspect-ratio 3D pillar electrodes. With this approach, a high pillar density can be achieved without compromising the aspect ratio, whereas reducing the pitch in systems fabricated with traditional cleanroom techniques would require sacrificing pillar height due to etching constraints.^44^ Furthermore, our streamlined process allowed the fabrication of micrometer-scale electrode sizes (3 and 6 μm in diameter), facilitating low-noise amperometric measurements. The functionality of these MEAs was confirmed through recordings from hiPSC-derived cortical organoids, detecting spontaneous activity and synchronized signals after 10 days of in vitro culturing. Although silver leakage was prevented by electroplating gold, alternative ink materials should be explored to avoid potential damage to organoids. Also, organoids measuring a few millimeters in size can develop a necrotic core, which should be addressed with vascularization strategies in the future.^45^ In addition, upcoming projects will focus on optimizing electrode pitch and material composition as well as exploring multifunctional electrode arrays for combined electrical and chemical recording. Overall, the results demonstrate the viability and potential for future applications in the monitoring of complex 3D cell cultures. In particular, we envision the application of our devices for electrochemical investigations within organoids.

## Methods

### Electrode Fabrication

Reagents were purchased from Sigma-Aldrich, USA, unless otherwise specified. 3-in. glass wafers (Borosilicate glass 3-in. glass wafer, MicroChemicals GmbH, Ulm, Germany; thickness: 500 μm) were cleaned using an ultrasonication bath (Bransonic ultrasonic cleaner 5510E-MTH, Branson ultrasonics, USA) for 5 min in acetone (VLSI Selectipur, BASF SE, Germany), 2-Propanol (99.5%) (IPA), and deionized (DI) water (Ultra Clear purification system/Berry Tec, Germany), respectively. Each 3-in. substrate yielded four MEAs. Lithography and lift-off procedures were employed to generate the electrode area, contact traces, and connection pads. A 50 nm thick layer of photoresist (Ma-N 1410, micro resist technology, Berlin, Germany) was spin-coated (Polo Spin 150i, Netherlands; 500 rpm for 10 s, followed by 3000 rpm for 25 s) on top of the cleaned substrate, exposed using a maskless aligner system (μMLA, Heidelberg Instruments Mikrotechnik GmbH, Heidelberg, Germany; dose: 160 mJ·cm^–2^, defoc: 0), kept for 3 min in developer (ma-D 533/s, micro resist technology, Berlin, Germany), rinsed with DI water, and dried in air. Afterward, a layer stack of 15 nm Ti and 100 nm of Au (5 × 10^3^ mbar argon, 12 W Au, 40 W Ti, Moorfield nanoPVD, UK) was sputtered on the glass slides. A lift-off step was performed to remove the remaining photoresist, immersing the glass wafers in a beaker filled with acetone and placing it in an ultrasonic bath (Bransonic ultrasonic cleaner 5510E-MTH, Branson ultrasonics, USA; 37 kHz, 100% power, 65 °C) until the remaining gold was fully removed.

3D pillars were fabricated on the samples using an advanced inkjet printer (CeraPrinter F-Series, Ceradrop, France) and silver nanoparticle ink (Silverjet DGP 40LT 15C, Sigma-Aldrich, USA). Prior to printing, the silver nanoparticle ink was sonicated for 30 min (Bransonic ultrasonic cleaner 5510E-MTH, Branson Ultrasonics, USA), filtered through a poly­(vinylidene fluoride) (PVDF) filter (GD/X, Whatman, Maidstone, UK; pore size: 0.45 μm), and loaded into a 2.4 pL cartridge (Samba, Fujifilm Dimatix, USA). A 40 V voltage pulse with rise, dwell, and fall times of 3, 10, and 1 μs, respectively, was used to eject single droplets onto the electrode pads. The sample stage and nozzle plate temperatures were set to 50 and 45 °C. Printing proceeded with a head speed of 55 mm·s^–1^, a drop-to-drop interval of 183 Hz, and droplet counts ranging from 500 to 2000. The deposition of 1000 droplets resulted in pillars with an average height of 471 ± 4 μm (*n* = 15, measured from one device). Completing the printing of two MEA arrays required roughly 3 h of unsupervised time. After printing, the Ag-pillar arrays were sintered thermally at 220 °C for 2 h. Samples were then rinsed with IPA and DI water (Ultra Clear purification system, Berry Tec, Germany).

For insulation, a 5 μm-thick layer of parylene C was deposited using chemical vapor deposition (SCS Labcoter 2, PDS 2010, Specialty Coating Systems, USA) from 3.15 g of dimer precursor (Daisan Kasei, Japan). Strips of polydimethylsiloxane (PDMS, Sylgard 184, 10:1 base/curing agent, Dow Corning, USA) were placed over the MEA contact pads before parylene C deposition and removed afterward to expose the connections. In the following step, all samples were sputtered with 15 nm of gold (30 mA, 8 × 10^–3^ mbar, Bal-tec MED 020, Liechtenstein) as preparation for the FIB milling process. Each MEA was mounted onto a specimen stub using double-sided conductive carbon tape and silver glue and stored at room temperature until the glue was fully cured. To create the openings in the pillar electrodes, a focused gallium ion beam (Crossbeam 550, Zeiss, Germany) was employed. The 2-step process involved milling (dose 1200 mC·cm^–2^ using 30 kV acceleration voltage, 1.5 nA beam current, circular milling pattern, spot diameter: 3 μm/6 μm, and depth 6 μm) followed by polishing (dose 200 mC·cm^–2^ using 30 kV acceleration voltage, 700 pA beam current, circular milling pattern, spot diameter: 1.5 μm/3 μm, and depth 1 μm).Quality control of the fabrication was conducted usingSEM­(SE2 detector, 3 kV acceleration voltage, 500 pA current) (Crossbeam 550, Zeiss, Germany) during and after the full procedure. The time required to process one pillar was approximately 10 min. To remove the thin gold layer, all samples were immersed in aqua regia solution (3:1 hydrochloric acid (HCl, 36%, BASF SE, Germany)/nitric acid (HNO_3_, 69%, BASF SE, Germany)) for 1 to 3 min until the gold was visibly removed.

Glass rings (15 mm in height, 17 mm outer diameter, and 14.6 mm inner diameter) were attached to the top of the MEA by dipping them in degassed PDMS and curing them on the sample for 1 h at 100 °C in an oven to contain the electrolyte solutions for electroplating of the pillar tips, MEA characterization, and organoid experiments.

Gold was electroplated onto the pillars as a protective measure to prevent silver leakage into the medium during both short-term and 10 day organoid experiments. The electrolyte employed was an aqueous potassium gold cyanide bath (KAu­[CN]_2_, Pur-A-Gold 401B, Enthone-OMI, The Netherlands). Electroplating was performed in a three-electrode setup using chronoamperometry with a potentiostat (VSP-300, Bio-Logic Science Instruments, France). In this setup, the 3D pillars served as the working electrode, a larger platinum mesh as the counter electrode, and an Ag/AgCl electrode (3 M NaCl, BASI, United Kingdom) acted as the reference. The reduction potential for KAu­[CN]_2_ was set to −1.15 V vs Ag/AgCl to achieve a filled tip. The gold was deposited with 200 cycles of 50 ms deposition time, followed by a 50 ms resting interval at 0.3 V for controlled deposition. After plating, all samples were rinsed with deionized water (Ultra Clear purification system/Berry Tec, Germany) and IPA, then dried in an oven at 70 °C for 1 h and stored at room temperature until further use. The material cost for a single MEA is in the range of 5 €, mainly determined by the substrate carrier.

### Imaging

A 3D laser scanning confocal microscope (VK-X250, Keyence, Japan) equipped with a 100× objective was used to capture images of the electrode area. The images were analyzed using MultiFile Analyzer software (Keyence, Japan), and the pillar height was determined. For SEM imaging, the samples were first coated with 15 nm of gold (30 mA, 8 × 10^–3^ mbar, Bal-tec MED 020, Liechtenstein). Each sample was mounted on SEM specimen stubs using conductive double-sided carbon tape and then imaged with a scanning electron microscope (Gemini 2, Zeiss Crossbeam 550, Germany) using a SE2 detector and a beam current of 500 pA. The acquired images were processed in GIMP by adjusting brightness and saturation and recoloring areas representing gold for enhanced visual clarity.

### Electrochemical Characterization

Cyclic voltammetry and impedance spectroscopy were carried out in PBS using a potentiostat (VSP-300, BioLogic Science Instruments, France) in a three-electrode configuration. This setup included an Ag/AgCl reference electrode (3 M NaCl, BASI, United Kingdom), a platinum coil wire as the counter electrode, and individual pillars as the working electrode. Cyclic voltammetry was performed over a potential range of −0.4 to 0.8 V with a scan rate of 50 mV·s^–1^ for 6 cycles. Impedance spectroscopy measurements were conducted by applying a sinusoidal signal with a 10 mV amplitude vs the reference electrode and frequencies ranging from 1 Hz to 10 kHz.

### Cortical Organoids

Penicillin/streptomycin, PBS, and cAMP were acquired from Sigma-Aldrich (USA). Geltrex Matrix, collagenase IV, and low attachment 6-well plates were bought from ThermoFisher Scientific (USA). Matrigel Matrix was purchased from Dow Corning (USA). l-ascorbic acid was purchased from Carl Roth (Germany). StemMACS iPS-Brew XF (human), dorsomorphin, SHH, SB 431542, BDNF, and GDNF were purchased from Miltenyi Biotec (Germany). 20% Knockout serum replacement, GlutaMAX, NEAA, 2-mercaptoethanol, DMEM/F12 (11330-032), N2 supplement, B27, and Neurobasal medium were bought from Life Technologies (USA). A-83 and CHIR 99021 were acquired from Tocris Bioscience (United Kingdom).

Cortical organoids were differentiated as previously described.^31^ In brief, hiPSCs (ISFi001 A; RRID:CVCL_YT30) were cultured on Geltrex-coated surfaces and maintained in human iPS Brew XF medium at 37 °C, 7% CO_2_ and 21% O_2_. Unless otherwise noted, the medium was changed daily. Once the colonies reached approximately 1.5 mm in size, they were detached using a 2 mg·ml^–1^ collagenase IV solution for 45–60 min and then incubated in iPS-Brew XF medium for 24 h in low-attachment 6-well plates on a 3D Rocker/shaker at 37 °C, 5% CO_2_, and 21% O_2_. The following day, the medium was replaced with the first forebrain-specific medium [20% Knockout Serum Replacement, 1× GlutaMAX, 1× NEAA, 0.02% 2-mercaptoethanol, 1× penicillin–streptomycin, 2 μM dorsomorphin, 2 μM A 83, 100 ng·ml^–1^ SHH in DMEM/F12]. On day 5, the medium was changed to the second forebrain-specific medium [1× N2 supplement, 1× GlutaMAX, 1× NEAA, 1× penicillin–streptomycin, 1 μM CHIR, 1 μM SB 431542 in DMEM/F12]. By day 7, the formed embryoid bodies (EBs) were embedded in Matrigel and cultivated in a Matrigel “cookie” for 5 days in the second forebrain-specific medium at 37 °C, 5% CO_2_, and 21% O_2_, but not on the 3D Rocker/shaker. On day 14, the organoids were released from the cookie using a 5 mL pipet tip. The medium was then replaced with the third forebrain-specific medium [1× N2 supplement, 1× B27, 1× GlutaMAX, 1× NEAA, 1× penicillin–streptomycin in DMEM/F12]. The organoids were transferred into low-attachment 6-well plates and incubated on a 3D Rocker/shaker at 37 °C, 5% CO_2_, and 21% O_2_, with medium changes every third day. On day 35, Matrigel (1:100) was added to the third forebrain-specific medium. At day 70, the medium was changed to the fourth forebrain-specific medium [1× B27 supplement, 1× GlutaMAX, 1× NEAA, 1× penicillin–streptomycin, 200 μM l-ascorbic acid, 500 μM cAMP, 20 ng·ml^–1^ BDNF, 20 ng·ml^–1^ GDNF in Neurobasal medium], with medium changes every fourth day.

### Extracellular Recordings

Before organoid placement, the 3D-pillar MEAs were sterilized by immersing them in ethanol for 2 h and then left to dry overnight under the cell culture bench. The organoids (age: 24 months) were carefully placed into the wells positioned above the 3D pillars of the MEAs using a cell strainer and a pipet tip, and fresh medium (DMEM/F12, 11330-032, Life Technologies, USA) was added. Extracellular signals were then recorded amperometrically with a custom-designed 64-channel amplifier, which was shielded within a Faraday cage. The amplifier was set to a 10 kHz sampling rate with a 1 GΩ feedback resistor, and recordings were performed using an Ag/AgCl reference electrode.

In total, we performed 111 measurements across 5 sessions (each session lasting between 30 and 60 min) over the course of 1 week. The individual measurements were performed on two MEAs with two organoids for a duration between 60 and 90s.

For 3 sessions of in situ measurements, the spontaneous activity of cortical organoids was monitored for a period of 5 to 10 min. After completing the recordings, the organoids were transferred back to the low-attachment 6-well plate, MEAs were rinsed with PBS, and negative control measurements in fresh media were performed. To prevent fouling, any remaining cell residue was properly removed from the 3D MEAs after the experiments using a 0.05% Trypsin–EDTA solution (Sigma-Aldrich, USA). The MEAs were incubated in the solution for 30 min, then rinsed with PBS, dipped in ethanol for 1 h, dried under the cell culture bench, and stored until further use. For 10 day experiments (2 sessions), the organoids were kept on the MEA, and the medium was replaced every second day. On day 1, one recording session was performed, where the spontaneous activity of cortical organoids was monitored. On day 10, both spontaneous and synchronized activity of the cortical organoids were recorded, after which the organoids were removed. The pillar MEAs were rinsed with PBS, fresh medium was added to the wells, and negative control measurements were conducted on the MEAs that were used. These measurements lasted approximately 45 to 60 min. Finally, the 3D MEAs were cleaned with a 0.05% Trypsin–EDTA solution (Sigma-Aldrich, USA) (incubation time: 30 min), rinsed with PBS, disinfected in ethanol for 1 h, and dried under the cell culture bench. Data analysis was performed using MATLAB (MathWorks, USA).

### Fluorescence Imaging

At the specified differentiation stage, organoids were collected, and the maintenance medium was discarded. The organoids were washed once with PBS and then fixed in formalin (F5554, 10%) for 20 min at 4 °C. Following fixation, organoids were washed three times with PBS and incubated in a 30% sucrose (S0389) cryoprotectant solution until fully saturated. The day after, organoids were placed in small embedding molds (7 mm × 7 mm) and equally spread if multiple organoids were embedded in one mold. The mold was filled with Neg-50 freezing medium (6502, Epredia, Germany) and stored at −80 °C. Once frozen, the organoids were sectioned into 40 μm thick horizontal slices at −20 °C using a freezing cryostat, and the sections were mounted onto slides.

### Immunostainings

PBS, BSA, Triton X-100, DAPI, CUX1 (SAB1405681), and TUBB3 (T5076) were acquired from Sigma-Aldrich (USA). NeuN (ab104224), GFAP (ab53554), BCL11B (ab18465), and TBR1 (ab31940) were bought from Abcam. Aqua-Poly/Mount was obtained from Polysciences Inc. (USA). Donkey-antimouse IgG Alexa 594 (A21203), Donkey-antirabbit IgG Alexa 594 (A21207), Donkey-antigoat IgG Alexa 488 (A11055), Donkey-antirat IgG Alexa 488 (A21208), and Donkey-antimouse IgG Alexa 488 (A21202) were bought from ThermoFisher Scientific (USA).

Immunostaining was carried out as described previously.^31,46^ The tissue sections were thawed at room temperature for 15 min. Blocking and permeabilization were performed by incubating the sections in a blocking solution [PBS containing 1% BSA and 0.3% Triton X-100] for 1 h at room temperature. Primary antibodies were diluted in the blocking solution, and incubation with primary antibodies was conducted overnight at 4 °C. Afterward, the sections were washed twice with PBS and incubated with secondary antibodies, diluted in a blocking solution (2 h at room temperature). Nuclei were counterstained with a 0.1 μg·ml^–1^ DAPI-PBS solution for 10 min at room temperature. Lastly, all sections were washed three times with PBS, and coverslips were mounted using Aqua-Poly/Mount. Primary antibodies were diluted as follows: CUX1 (1:1000), TUBB3 (1:1000), TBR1 (1:1000), BCL11B (1:1000), NeuN (1:500), GFAP (1:1000). Secondary antibodies were diluted 1:500.
